# Measuring the impact of chronic conditions and associated multimorbidity on health-related quality of life in the general population in Hong Kong SAR, China: A cross-sectional study

**DOI:** 10.1371/journal.pone.0224970

**Published:** 2019-11-20

**Authors:** Eliza Lai yi Wong, Richard Huan Xu, Annie Wai ling Cheung

**Affiliations:** The Jockey Club School of Public Health and Primary Care, The Chinese University of Hong Kong, Hong Kong SAR, China; Sciensano, BELGIUM

## Abstract

**Objectives:**

The aims of this study were to 1) evaluate the impact of eight common chronic conditions and multimorbidity on preference-based health-related quality of life (HRQoL), and 2) estimate the minimally important difference (MID) in the general population of Hong Kong (HK).

**Design:**

Data were analyzed using secondary data analysis based on a cross-sectional, population-based validation study of HK’s general population.

**Participants:**

A representative sample was recruited across eighteen geographical districts in HK, and 1,014 HK Chinese residents aged 18 years and older participated in the survey. The prevalence of chronic conditions among the respondents was 30.3%.

**Interventions:**

The HRQoL was assessed using the locally validated version of EQ-5D-5L. The five-dimension descriptive system, and the utility scores of EQ-5D-5L were used as the dependent variable in the study. Eight common chronic conditions, multimorbidity, and demographic characteristics were defined as predictors in the analysis. Chi-squared test, analysis of variance (ANOVA), logistic regression, and Tobit regression models were used to analyze the data. A simulation-based approach was used to calculate the MID based on instrument-defined single level transitions.

**Results:**

The findings indicated that respondents with physical disabilities were more likely to report problems on all five dimensions of the EQ-5D-5L than those with other chronic conditions. In addition, respondents with multiple chronic conditions were more likely to report health problems and lower utility scores of EQ-5D-5L. The mean of MID estimates among the respondents in HK was 0.093 (standard deviation = 0.001), which is higher than in other Asian countries.

**Conclusions:**

The findings suggest that having more chronic conditions is strongly associated with a lower HRQoL. Healthcare reforms to address foreseeable challenges arising as more patients live with chronic conditions and multimorbidity could improve the HRQoL of HK citizens.

## Introduction

Chronic condition is described as a health problem that persists across time and requires some degree of health care management [1]. Globally, chronic conditions are on the rise, leading to diminished health-related quality of life (HRQoL), reduced physical functioning, and a higher risk of depression and anxiety [2–4]. Multimorbidity is commonly defined as the presence of two or more chronic conditions in an individual [5]. Given that most healthcare systems worldwide were designed to focus on single-disease management, multimorbidity poses major challenges for healthcare providers [6]. There is a consensus that current disease-specific clinical guidelines might be inappropriate for patients with more than one chronic conditions. If we follow the current guidelines, and each condition is considered in isolation, patients will have to visit healthcare providers more frequently, implement life-style changes and take more medicines [7]. In view of the complexity of efficiently caring for patients with chronic condition and multimorbidity, there is now a global consensus that a key component of patient-centered healthcare should be support specifically designed to manage multimorbidity. Healthcare services should transfer the focus from the deterioration of physically functioning caused by a chronic condition or associated multimorbidity to the proactive improvement of patients’ HRQoL.

Chronic conditions, especially multimorbidity, have the potential to induce profound negative effects on a person’s HRQoL or wellbeing, which has been the subject of studies for decades [5,8,9]. Recently, in Asian-Pacific countries, a growing number of studies has indicated an inverse relationship between multimorbidity and HRQoL in Australians [10] and Korean adults, especially elderly women with a lower socioeconomic status [11]. In addition, Pati et al. found a significantly high impairment of physical and mental HRQoL in patients with multimorbidity above the age of 50 years and different combinations of chronic conditions appears to impact on HRQoL differently [12]. However, although an increasing numbers of people in Hong Kong (HK) live with multimorbidity, few studies explored the effect of multimorbidity on the HRQoL using generic preference-based measure in that population. A study by Chung et al. of the HK general population demonstrated the following significant risk factors for chronic conditions and multimorbidity: being female and 25 years or older, having a low education level, having a low household income, and being unemployed or retired [13]. Another recent study conducted by Chin et al., which used the SF-12 questionnaire with patients from primary care clinics in HK, found a positive relationship between multimorbidity and depressive symptoms [14]. Although these studies are valuable and heuristic, did not explore the impact of multimorbidity on HRQoL in the local population. Understanding this impact could enable policymakers to design effective and patient-centered healthcare system to address the growing challenge of multimorbidity.

An increasing number of studies conducted worldwide have explored new approaches to evaluate HRQoL [15], in recent decades, several instruments have been introduced in this field. For example, the EQ-5D, developed by the EuroQol group, is a simple and generic measurement that is widely applied for HRQoL analysis [16], which is increasingly used as a guideline to inform the evaluation of patient-reported outcomes in many jurisdictions [15]. Recently, there has been an increased interest in defining and calculating the minimally important difference (MID) of utility scores using generic measurement. MID could be used to quantify the minimal variation in an index score, such as the EQ-5D, and represents a meaningful improvement for the patient in order to help professionals assess patient-reported outcomes based on different diseases or conditions. Around the world, several countries, including Canada, China, Spain, Japan, England, and Uruguay, have established the MID for their general populations [17]. However, no MID estimates have been published using the EQ-5D-5L scoring algorithms for the HK general population. Therefore, the aims of this study were to 1) evaluate the impact of eight common chronic conditions and multimorbidity on HRQoL, and 2) estimate the MID in HK’s general population.

## Method

### Study design and data collection

The study was conducted using the data derived from the valuation study of the preference-based health index using the HK EQ-5D-5L in HK [18], through a cross-sectional and population-based survey using the locally validated EQ-5D questionnaire (EQ-5D-5L HK). In that study, a survey was conducted among HK Chinese residents aged 18 years and older. The study recruited a representative sample using the stratified quota method in terms of sex, age, and highest level of educational attainment from all eighteen geographical districts of HK. The composition of the final sample of respondents was comparable to the HK general population [19]. Face-to-face interviews were conducted with the aid of computer-based valuation software (The EuroQol Valuation Technology, EQ-VT) [20]. During those interviews, the respondents self-reported their HRQoL using the EQ-5D-5L which includes a descriptive system of 5 health status dimensions and an overall health rating scale- Visual Analogue Scale (EQ-VAS), and socioeconomic information data, including: age, sex, marital status, educational level, as well as their experience with chronic conditions. Ethical approval of original study was obtained from the Joint Chinese University of Hong Kong–New Territories East Cluster Clinical Research Ethics Committee. Written informed consent forms were obtained from all respondents.

### Multimorbidity

During the interview, respondents were asked, ‘Do you have any kind of chronic condition?’ If the respondent answered ‘yes’, respondents were then asked to self-report their health status whether having the following conditions: deafness or severe hearing impairment, blindness or partially sighted, a long-standing physical disability, a learning problem, a mental health condition, or any chronic illnesses. All the conditions must be formally diagnosed by the healthcare professionals. Each chronic condition was coded as present or absent in the analysis. Using the count method, multimorbidity was defined as two or more chronic conditions occurring simultaneously [10].

### Instrument–EQ-5D-5L HK

The standard EQ-5D-5L consists of self-reported health states on a five-dimension descriptive system and a self-reported overall health rating using EQ-VAS. The descriptive system comprises five dimensions: mobility, self-care, usual activities, pain/discomfort, and anxiety/depression. Each dimension in the EQ-5D-5L has five response options, ranging from 1 to 5: no; slight; moderate; severe; and extreme problems. The reported health states of the five dimensions can be converted into a single health index (utility score) using a scoring algorithm based on cultural health preferences. The utility score ranges between 0 and 1, where 1 represents ‘full health’, 0 represents ‘death’, and negative values represent ‘worse than death’. The EQ-5D-5L HK version was developed and validated following the latest international protocol, ensuring the evaluation of people’s HRQoL considering their perceptions in the context of HK’s cultural and value systems [18]. The EQ-5D-5L HK was used to measure the respondent HRQoL of the respondents, after which the utility score of EQ-5D-5L was derived using the established HK value set [18] by weighting each respondent’s self-reported health states based on a single preference-based health index (utility scores). The normative profile of HRQoL for HK’s general population has been also reported [21].

### Statistical analysis

R (R Foundation, 2019, version: 3.5.1) was used for data analysis. The variables for background characteristics were regrouped for analysis. Age was divided into four groups: 18–24, 25–44, 45–64, and ≥ 65 years. Education level was categorized into three groups: primary/below, secondary/sub-degree, and postsecondary/degree. Employment status was used as a proxy for an individual’s economic status and grouped as follows: retired, non-employed and employed. Living conditions were registered as either living alone or with family. Marital status was a multi-status grouping: single, separated/divorce/widowed, and married. Household type was grouped into two categories: self-owned and tenant. Means and standard errors of the EQ-5D index using the HK value set were calculated and presented separately by sex, age, education, and other background characteristics. The percentages of people reporting problems in each dimension was calculated according to sex, age and educational levels. To test the statistical significances of the differences between groups having reported problems in different dimensions of the health states and EQ-5D utility scores, Chi-square tests and ANOVA were conducted.

Binary logistic regression models were used to identify the predictive characteristics of the respondents, such as whether different chronic conditions and multimorbidity status (number of problems in the five EQ-5D dimensions) predicted problems in each EQ-5D dimension (respondent reported having any problem on EQ-5D was defined as having problem, code as 1; respondent reported having no problem on EQ-5D was defined as having no problem, code as 0), while controlling for covariates such as age, sex, and educational attainment. A Tobit regression model (regression for censored data) was used to estimate and predict EQ-5D utility scores for respondents with different demographics and self-reported health states (package AER in R). Pairwise deletion of missing data was adopted in the analysis. The statistical significance was set at *p* ≤ 0.05 for all analyses using two-sided tests.

The MID was calculated based on the average absolute difference between the utility score of the baseline health state and the utility score of all single-level transitions from the baseline health state. Details about the concept of MID can be found in Pickard et al.’s work [22]. A simulation-based approach based on instrument-defined single-level transitions was adopted to provide supportive information for the MID estimation among HK population. According to McClure’s suggestions, the adjusted MID was further suggested to estimate by excluding the maximum value of single-transitions within different levels of EQ-5D dimensions in order to diminish the bias [23]. In the HK scoring algorithm, the transition between level 3 (moderate problem) and level 4 (extreme problem) in each dimension is a maximum-valued scoring parameter than the transitions between any other levels. Thus, the adjusted MID among HK population was estimated by excluding the transition between level 3 and level 4 within each dimension.

## Results

A total of 1,033 HK residents participated in the study. The results of 19 respondents were discarded after either declining to be interviewed due to unavailability or after providing incomplete responses, leaving 1,014 responses for data analysis. Table 1 shows the background characteristics of the sampled respondents. The percentage of women in the study was 59.2%. An education level below primary school was reported by 19.8% of respondents, and nearly 70% of respondents were between the ages of 25 and 65 years. The prevalence of chronic conditions and multimorbidity was 30.3% and 12.3%, respectively. No statistically significant differences were found between our sample and the general population in terms of background characteristics and chronic conditions.

**Table 1:** Background characteristics of respondents.

	Sample					General population^##^ %	p-value^###^
	N	%	Mean^#^ (Range)	SE	VAS		
**Overall**	1014	100	0.918 (0.022–1.0)	0.004	82.72		
**Sex**							
Male	414	40.8	0.919	0.006	81.81	46.0	0.07
Female	600	59.2	0.917	0.005	83.34	54.0	
**Age(mean = 45.67 years)**							
18–24	166	16.3	0.938	0.005	80.81	10.7	0.24
25–44	346	34.1	0.938	0.004	82.98	30.4	
45–64	342	33.7	0.905	0.007	83.42	31.7	
> = 65	160	15.8	0.883	0.015	82.62	15.9	
**Education**							
Primary/below	201	19.8	0.868	0.006	82.06	14.6	0.31
Secondary	615	60.7	0.927	0.003	83.20	47.3	
Post-secondary	198	19.5	0.904	0.003	81.88	32.7	
**Living condition**							
Live alone	75	7.4	0.874	0.006	80.92	NA	
Live with family	939	92.6	0.922	0.004	82.86		
**Employment**							
Retired	193	20.4	0.884	0.005	81.65	NA	
Non-employed	386	40.8	0.916	0.004	83.3		
Employed	368	38.9	0.938	0.003	82.5		
**Marital status**							
Single	322	31.8	0.932	0.003	80.61	30.1	0.91
Married	583	57.5	0.916	0.004	83.91	58.4	
Divorced/Separated/Widow	109	10.7	0.893	0.005	82.50	5.1	
**Household**							
Self-owned	484	47.7	0.925	0.003	82.76	NA	
Tenant	530	52.3	0.913	0.004	82.70		
**Chronic conditions**							
Yes	307	30.3	0.871	0.01	81.15	28.4	0.33
No	707	69.7	0.942	0.01	83.40	72.6	
**Number of multimorbidity**							
0	707	69.7	0.94	0.01	83.41		
1	183	18.0	0.88	0.01	81.76		
2	95	9.4	0.86	0.02	81.10		
> = 3	29	2.9	0.73	0.05	74.38		

# Mean and SE = EQ-5D utility’s mean and SE; the bracket indicated the range of EQ-5D utility in this sample (min~max)

## Hong Kong census 2016, https://www.bycensus2016.gov.hk/en/

### Chi-squared test was used to generate the p-value

**Fig 1:**
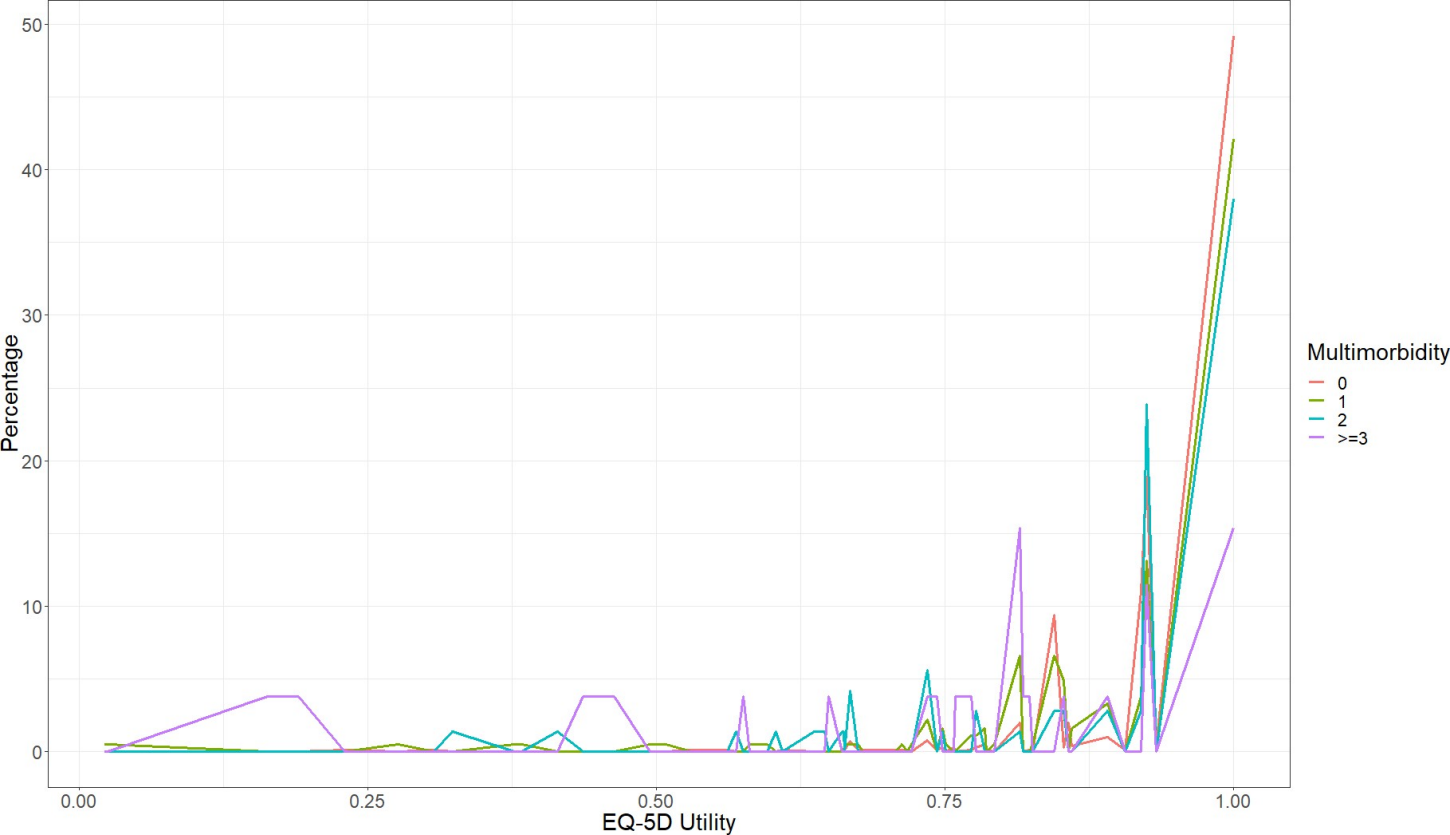
The distribution of EQ-5D utility of multimorbidity.

**Fig 2:**
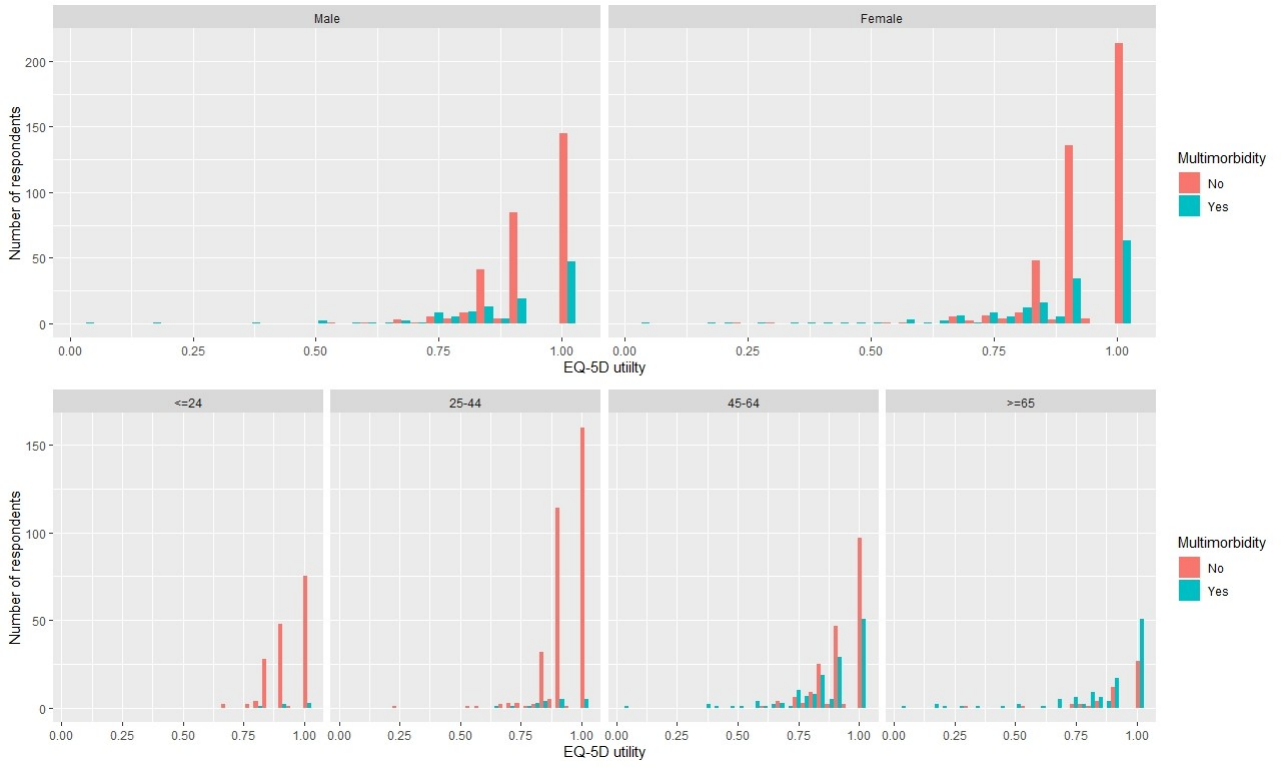
The distribution of EQ-5D utility of gender and age group based on people’s chronic conditions.

**Fig 3:**
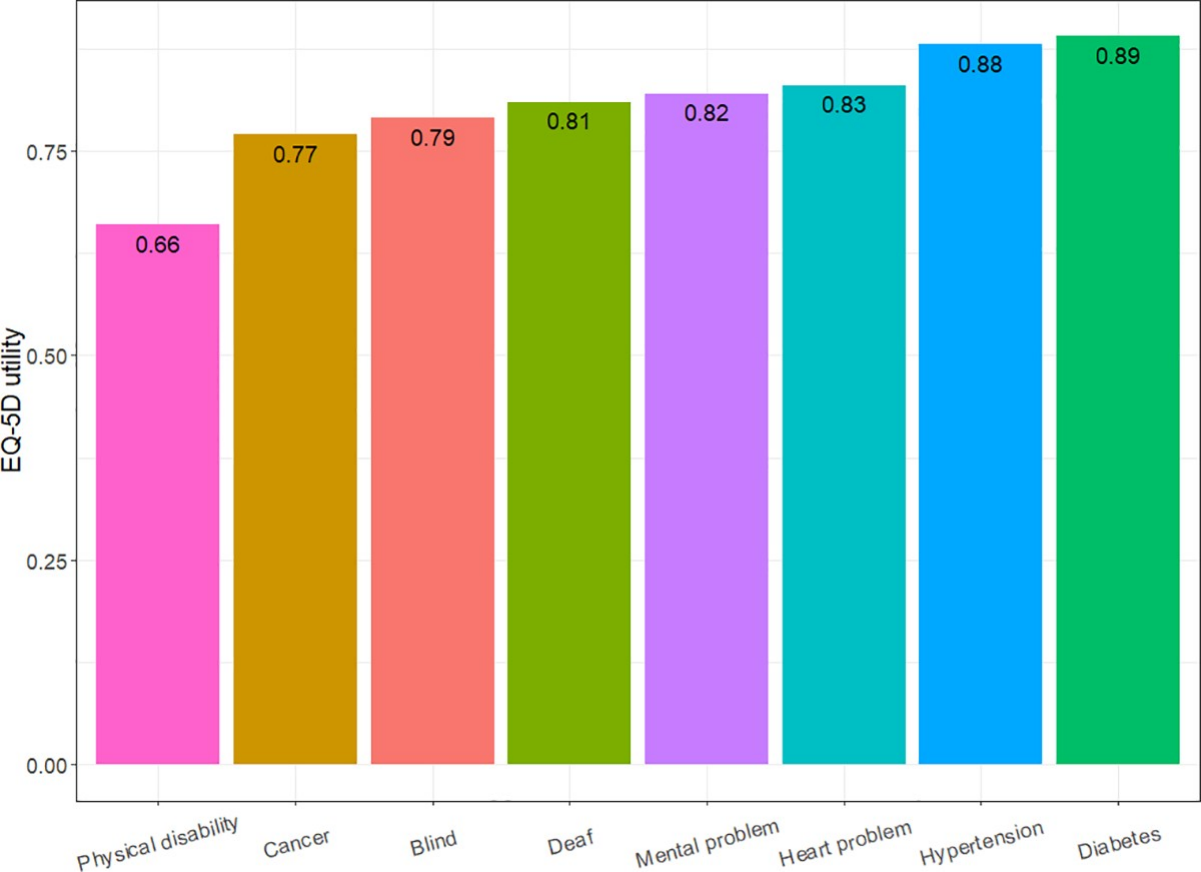
The EQ-5D mean utility for each chronic condition.

Fig 1 indicates that nearly 51% of respondents with no chronic conditions reported full health (utility = 1.0), 39.4% with one chronic condition, 36.9% with two chronic conditions, and 14.7% with three or more chronic conditions also reported full health. The health utility began to fluctuate when there were two or more chronic conditions. Fig 2 shows the distribution of EQ-5D utility by sex and age groups based on the respondents’ chronic conditions (with/without multimorbidity). The distribution of the EQ-5D utility scores for both men and women was similar and more respondents reported no multimorbidity scored a higher EQ-5D utility than respondents with multimorbidity. Fewer respondents (3.8% and 3.0% respectively) with multimorbidity in younger groups (18–24 years and 25–44 years) reported having full health (utility = 1.0). Lower utility scores were reported for respondents with multimorbidity than those without multimorbidity in all age groups, except for the oldest group (aged 65 and above). 65.4% of respondents with multimorbidity aged 65 years and above reported full health. Fig 3 depicts the mean utility score of EQ-5D for each chronic condition. Respondents with diabetes had the highest utility score of 0.89, followed by, hypertension, heart disease, and mental problems. Physical disability scored the lowest score with 0.66.

Table 2 shows the percentage of respondents with health problems on EQ-5D reporting multimorbidity by sex, age and educational level. Findings showed that elderly respondents were more likely to have multimorbidity. 75% of all respondents aged 65 years or older with three or more chronic conditions reported having problems with self-care, and follow by anxiety/depression (66.7%), mobility (62.5%), pain/discomfort (61.1%), and usual activities (55.6%). Moreover, up to 76.9% of the respondents with two chronic conditions between 45 and 64 years of age reported having some problems with regular daily activities. Respondents with a low educational level tended to report having multimorbidity on all five dimensions of EQ-5D, remarkably, 100% of respondents with ≥ 3 chronic conditions reported having problem on self-care were not received any education or only completed primary school. No statistically significant difference was identified between male and female respondents.

**Table 2:** Percentage of respondents with health problem on EQ-5D reporting multimorbidity by sex, age group and educational level.

	Mobility (%)				Self-care (%)				Usual activities (%)				Pain/discomfort (%)				Anxiety/ depression (%)			
	0	1	2	> = 3	0	1	2	> = 3	0	1	2	> = 3	0	1	2	> = 3	0	1	2	> = 3
**Total**	36.97	34.45	15.13	13.45	20.00	33.33	20.00	26.67	44.83	29.89	14.94	10.34	66.42	19.71	9.50	4.40	68.94	21.21	6.44	3.41
**Age group**																				
18–24	13.64	0	0	0	0	0	0	0	23.08	3.85	0	0	21.98	2.47	0	0	26.92	1.79	0	0
25–44	29.55	4.88	0	6.25	66.67	0	0	0	23.08	11.54	0	11.11	41.39	11.11	0	5.56	46.7	16.07	0	0
45–64	45.45	58.54	50	31.25	33.33	40	66.67	25	43.59	61.54	76.92	33.33	28.94	58.02	58.97	33.33	21.43	60.71	58.82	33.33
> = 65	11.36	36.59	50	62.50	0	60	33.33	75	10.26	23.08	23.08	55.56	7.69	28.4	41.03	61.11	4.94	21.43	41.18	66.67
**P-value**	**<0.001**				0.082				**0.006**				**<0.001**				**<0.01**			
**Sex**																				
Male	50	43.9	33.33	37.5	0	40	33.33	25	35.9	42.31	7.69	44.44	41.76	44.44	23.08	44.44	40.11	46.43	29.41	44.44
Female	50	56.09	66.67	62.5	100	60	66.67	75	64.1	57.69	92.31	55.56	58.24	55.56	76.92	55.56	58.89	53.57	70.59	55.56
**p-value**	0.626				0.655				0.153				0.126				0.63			
**Education**																				
Primary/below	25.0	42.50	73.67	68.81	0	80.0	66.7	100	28.21	36.0	64.29	77.78	12.45	34.18	53.66	72.22	0.082	30.91	72.22	77.78
Secondary	65.89	57.50	26.33	25.0	100	20.0	33.3	0	53.85	56.0	35.71	11.11	67.77	56.96	46.34	22.22	62.64	54.55	27.78	11.11
Post-secondary	9.11	0	0	6.19	0	0	0	0	17.95	8.0	0	11.11	19.78	0.088	0	0056	29.12	14.54	0	11.11
**p-value**	**0.002**				**0.04**				**0.03**				**<0.001**				**<0.001**			

0 = no chronic conditions; 1 = one chronic condition; 2 = two chronic conditions; > = 3 = equal or more than three chronic conditions.

Table 3 presents the relationships between chronic conditions and EQ-5D health states in each dimension, with adjustments for background characteristics (sex, age, and educational attainment). Findings showed that respondents with physical disabilities were most likely to report having health problems on all five dimensions of EQ-5D, especially for self-care (OR = 30.78, 95% C.I [5.15, 94.51], *p* < 0.001). The Tobit model revealed that respondents with physical disabilities (beta = -0.28), mental problems (beta = -0.14), hypertension (beta = -0.05), or cancer (beta = -0.18) were significantly associated with having lower EQ-5D utility scores. Moreover, people living with multimorbidity were more likely to report health problem on all five dimensions of EQ-5D. For example, respondents living with three or more chronic conditions were 19 times more likely to have problems with self-care than those living without chronic conditions, and more likely to have problems with mobility (OR = 12.09), usual activities (OR = 7.59) and pain/discomfort (OR = 3.59). The Tobit model also revealed that respondents living with an increasing number of chronic conditions (one condition [beta = -0.045], two [beta = -0.046], and ≥three [beta = -0.187]) tend to have significantly lower utility scores.

**Table 3:** The chronic conditions and multimorbidity model on EQ-5D utility and its five dimensions.

	Mobility OR(95% CI)		Self-care OR(95% CI)		Usual activities OR(95% CI)		Pain/discomfort OR(95% CI)		Anxiety/depression OR(95% CI)		EQ-5D utility β(95% CI)	
	Model I #&	Model II	Model III	Model IV	Model V	Model VI	Model VII	Model VIII	Model IX	Model X	Model XI	Model XII
**Sex**												
Male	Ref	Ref	Ref	Ref	Ref	Ref	Ref	Ref	Ref	Ref	Ref	Ref
Female	0.78 (0.49,1.22)	0.78 (0.51,1.21)	1.83 (0.49,8.76)	1.68 (0.52,6.55)	1.33 (0.80,2.24)	1.36 (0.83,2.25)	0.75 (0.44,1.28)	0.99 (0.76,1.29)	0.98 (0.73,1.33)	1.02 (0.75,1.37)	0.004 (-0.02,0.029)	0.001 (-0.014,0.015)
**Age(years)**												
18–24	Ref	Ref	Ref	Ref	Ref	Ref	Ref	Ref	Ref	Ref	Ref	Ref
25–44	1.11 (0.43,3.03)	1.17 (0.46,3.37)	5.53 (0.02,17.55)	6.26 (0.21,8.26)	0.47 (0.19,1.15)	0.49 (0.21,1.21)	0.99 (0.75,1.29)	0.89 (0.60,1.32)	0.84 (0.55,1.28)	0.84 (0.56,1.29)	0.009 (-0.027,0.045)	0.004 (-0.017,0.026)
45–64	2.15 (0.91, 3.21)	2.23 (0.93,6.21)	3.31 (0.09,14.72)	5.83 (0.69,12.21)	1.08 (0.48,2.56)	1.13 (0.50,2.69)	0.88 (0.59,1.32)	1.04 (0.68,1.59)	0.64 (0.41,1.03)	0.63 (0.39,1.02)	0.009 (-0.03,0.047)	-0.001 (-0.024,0.022)
> = 65	2.05 (0.75, 6.19)	1.94 (0.72,5.88)	3.21 (0.21,9.34)	6.27 (0.39,9.95)	0.46 (0.16,1.34)	0.46 (0.16,1.32)	1.01 (0.67,1.54)	0.79 (0.46,1.38)	**0.42** (0.22,0.79)^**^	**0.41** (0.22,0.77)^**^	0.036 (-0.014,0.086)	0.013 (-0.017,0.043)
**Education**												
Primary/below	Ref	Ref	Ref	Ref	Ref	Ref	Ref	Ref	Ref	Ref	Ref	Ref
Secondary/Sub-degree	**0.50** (0.29,0.85)^*^	**0.57** (0.34,0.93)^*^	0.39 (0.09,1.67)	0.40 (0.10,1.43)	**0.38** (0.21,0.70)^**^	**0.46** (0.26,0.81)^**^	0.79 (0.46,1.38)	0.89 (0.61,1.31)	0.78 (0.51,1.23)	0.83 (0.54,1.29)	**0.059** (0.024,0.094)^***^	**0.036** (0.015,0.057)^***^
Post-secondary/Degree	**0.18** (0.05,0.48)^**^	**0.20** (0.06,0.53)^**^	0.26 (0.02,3.59)	0.23 (0.03,1.48)	**0.39** (0.15,0.97)^*^	0.48 (0.19,1.14)	0.88 (0.60,1.29)	0.63 (0.38,1.03)	1.04 (0.60,1.81)	1.12 (0.65,1.92)	**0.068** (0.022,0.113)^**^	**0.041** (0.014,0.068)^**^
**Chronic conditions**												
Deafness	3.79 (0.91,15.1)		2.60 (0.07,39.66)		0.41 (0.04,2.64)		1.00 (0.27,3.56)		0.96 (0.20,3.72)		-0.036 (-0.144,0.073)	
Blindness	0.79 (0.22,2.43)		1.08 (0.05,11.72)		2.23 (0.63,6.80)		1.25 (0.49,3.17)		1.79 (0.66,4.64)		-0.063 (-0.144,0.017)	
Physical disability	**22.89** (8.15,75.51)^***^		**30.78** (5.16,194.51)^***^		**9.24** (3.40,24.83)^***^		**3.29** (1.28,9.54)^*^		**3.73** (1.50,9.27)^**^		**-0.279** (-0.355,-0.203)^***^	
Mental problem	**3.48** (1.34,8.36)^**^		6.99 (0.62,44.81)		**4.21** (1.54,10.44)^**^		1.40 (0.66,2.98)		**2.88** (1.33,6.19)^**^		**-0.136** (-0.201,-0.071)^***^	
Heart disease	2.02 (0.79,4.83)		4.64 (0.71,23.73)		1.38 (0.43,3.82)		**2.34** (1.10,5.25)^*^		0.84 (0.32,1.98)		-0.059 (-0.124,0.007)	
Hypertension	**2.04** (1.19,3.50)^**^		**7.86** (1.82,42.11)^**^		**1.88** (1.01,3.49)^*^		1.22 (0.81,1.82)		1.55 (0.97,2.46)		**-0.045** (-0.081,-0.008)^*^	
Diabetes	0.99 (0.51,1.86)		**0.06** (0.01,0.57)^*^		0.79 (0.34,1.72)		1.18 (0.69,1.99)		0.60 (0.31,1.14)		0.018 (-0.031,0.066)	
Cancer	1.76 (0.37,6.36)		5.13 (0.25,38.42)		**4.10** (1.02,14.09)^*^		3.19 (1.01,11.94)		3.00 (0.93,9.33)		**-0.183** (-0.282,-0.085)^***^	
**Multimorbidity**												
No		Ref		Ref		Ref		Ref		Ref		Ref
1		**2.65** (1.57,4.49)^***^		3.76 (0.79,21.94)		**2.33** (1.26,4.27)^**^		1.24 (0.86,1.81)		**1.84** (1.21,2.79)^**^		**-0.045** (-0.065,-0.024)^***^
2		**2.61** (1.29,5.14)^**^		4.65 (0.71,33.35)		**2.74** (1.23,5.93)^*^		**1.84** (1.07,3.19)^*^		1.41 (0.73,2.63)		**-0.046** (-0.076,-0.016)^**^
> = 3		**12.09** (4.89,31.22)^***^		**19.16** (3.18,134.77)^**^		**7.58** (2.75,20.27)^***^		**3.58** (1.51,9.22)^**^		**2.46** (1.01,5.91)^*^		**-0.187** (-0.234,-0.141)^***^

# Model I = mobility chronic condition model; Model II = mobility multimorbidity model; Model III = self-care chronic condition model; Model IV = self-care multimorbidity model

Model V = usual activities chronic condition model; Model VI = usual activities multimorbidity model; Model VII = pain/discomfort chronic condition model; Model VII = pain/discomfort multimorbidity model; Model IX = anxiety/depression chronic condition model; Model X = anxiety/depression multimorbidity model; Model XI = EQ-5D utility chronic condition model; Model XII = EQ-5D utility multimorbidity model

& Model I, II, III, IV, V, VI, VII, VIII, IX, and X were applied with binary logistic regression model; Model XI and Model XII were applied with Tobit regression model

* *p*<0.05

** *p*<0.01

*** *p*<0.001

Table 4 shows the results of our MID estimates among HK general population. Both the mean and median MID estimate was 0.093 (S.D. of mean = 0.011, interquartile range IQR of median: 0.085 ~ 0.101). The maximum-valued scoring difference was 0.189, referring to the transition between level 3 (moderate) and level 4 (severe) of mobility. The minimum-valued scoring difference was 0.026, referring to the transition between level 2 (minor) and level 3 (moderate) of self-care dimension. Moreover, the adjusted mean MID was 0.071 (S.D. = 0.008). For respondents living with chronic conditions, the mean MID estimate was 0.083 (S.D. = 0.006) while the adjusted MID estimate was 0.079 (S.D. = 0.006).

**Table 4:** MID estimate of EQ-5D-5L utility score using HK scoring algorithm.

Score diff	Level 1 vs. Level 2	Level 2 vs. Level 3	Level 3 vs. Level 4	Level 4 vs. Level 5	Mean for MID (SD)	Adjusted MID (SD)	Median for MID (interquartile range IQR)
**For general population**							
**MO**	0.109	0.073	0.189	0.158	0.093 (0.011)	0.071(0.008)	0.093 (0.085–0.101)
**SC**	0.087	0.026	0.158	0.081			
**UA**	0.067	0.027	0.140	0.048			
**PD**	0.076	0.071	0.160	0.047			
**AD**	0.080	0.060	0.153	0.055			
**For patients with chronic conditions**					0.083 (0.006)	0.079 (0.006)	0.084 (0.081–0.084)

MO = mobility, SC = self-care, UA = usual activity, PD = pain/discomfort, and AD = anxiety/depression

Level 1 = no problem; Level 2 = with minor problem; Level 3 = with moderate problem; Level 4 = with severe problem; Level 5 = with extreme problem/ unable to

## Discussion

This study demonstrated the impact of chronic conditions and associated multimorbidity on HRQoL in HK’s adult general population. Overall, people reported having physical disability had lower EQ-5D utility scores than those with other chronic conditions. There was a significant association between multimorbidity and decreasing HRQoL, when controlling for socio-demographic characteristics. Our results are generally consistent with findings from previous studies in UK, France, Australia, the US [8,10,24,25] and other Asian countries, such as Singapore and Japan [26,27]. Moreover, our mean MID estimate of EQ-5D-5L for HK’s general population was 0.093 (adjusted MID = 0.071).

Our findings showed that HRQoL was significantly and negatively affected by the number of chronic conditions and the type of condition, particularly, involving physical disability. Previous studies have indicated similar findings in terms of the associations between physical functioning and HRQoL. for example, in a longitudinal study in Norway, Tollisen et al. found a strong correlation between physical disability and HRQoL in juvenile idiopathic arthritis patients [28]. Further, in a French study, Mouthon et al, found that patients with systemic sclerosis with reduced wrist and hand mobility had lower mental HRQoL [29]. It is important for policymakers to consider improving HRQoL by incorporating additional assistance measures in public spaces and providing barrier-free access throughout communities and natural environments, empower people with physical disabilities to live well in communities, which can improve their HRQoL. However, these findings should be interpreted with some caution. Physical functional impairments may impact respondents’ psychological status, and psychological instability may lead to physical discomfort, both of which could potentially lead to fluctuations of HRQoL [30–32]. However, our study was unable to distinguish between the fluctuations of HRQoL due to physical activity engagement and those due to psychological health improvement or deterioration. It should be further explored in the future.

Our study indicated that people living with multimorbidity were more likely to report worse HRQoL. The management of multimorbidity is complex, caused primarily by the challenges of specialized healthcare, which results in fragmented care, polypharmacy, multiple treatment burdens, mental health problems, and increased healthcare utilization that strains available resources [33–35], which was described by O’Brien as an “endless struggle” for comorbidity patients [36]. When dealing with increased threats from multimorbidity, two priorities need to be addressed by government and professional entities. First, chronic condition management should target functions and abilities necessary to manage daily household and social activities, not only the disease itself [4]. Second, revisions to clinical guidelines and research protocols are required because they currently focus only on care programs for single disease. The traditional clinical guidelines might be appropriate for patients living with a single condition, but the sum of clinical recommendations from various sources suggest different approaches for multimorbidity [37,38]. Further studies should explore the influence of different combinations of chronic conditions on HRQoL. Those findings could provide added value when designing guidelines for different chronic conditions which might be grouped or separately assessed. These predictions could be extremely useful for front-line medical staff seeking optimal approaches to care for patients with multimorbidity.

The study also found that educational level has a significant impact on the relationship between the EQ-5D utility score and multimorbidity. Some previous studies have reported that people with low educational level tend to have low HRQoL. For example, a study in Germany found that adults in low educational groups experienced increased levels of health impairments as well as lower HRQoL once health is impaired [39]. Another study that investigated esophageal cancer patients’ HRQoL in Sweden found that low education was associated with poorer functioning in certain HRQoL domains for women [40]. Similarly, a recent study in Malaysia that included 347 respondents in a low household income group found that the quality of life (QoL) was negatively associated with educational level and chronic conditions. However, few studies have explored the relationship between HRQoL and levels of education in the context of people with multimorbidity. Considering similar findings our previous study in specialist out-patient clinics in HK [41], we believe that in HK, people with a low educational level and multimorbidity have lower EQ-5D utility. One reason for this finding is that education can provide skills and knowledge that are essential for navigating the complex healthcare system. As educational inequity is intertwined with the expanding epidemic of multimorbidity [38], policymakers should pay close attention to these linked phenomena.

Additionally, age is not necessary inversely associated with multimorbidity patients’ HRQoL. In fact, elderly respondents, in our sample were less likely to report depression or anxiety than young respondents. Previous studies have similarly found that elderly patients are more likely to develop better resilience and adaptability to living with multimorbidity through their life experiences and wisdom, thus resulting in higher self-reported HRQoL [42]. However, an essential question must be asked: Does high utility score really reflect a better life for this population? Further, does better health equate to higher HRQoL? Apparently, the answer is not simply yes or no. Discussing the limitations of EQ-5D is beyond the scope of this paper (Brazier has discussed this in depth [43]). However, when we discuss people’s HRQoL, specifically those living with either a single chronic condition or multimorbidity, this study suggests HRQoL is impacted by things other than chronic conditions or multimorbidity. Otherwise, young people should have higher HRQoL than elderly people. Instead, factors such as equity, fairness, and public willingness might also have strong influences on well-being [44]. More effort and resources should be invested into developing valid and reliable instruments to evaluate people’s overall QoL, rather than just HRQoL [43].

Although the evidence is limited, the estimation of MID using generic preference-based measure is highly recommended to provide insight on the evaluation of clinical interventions from the perspective of HRQoL [45]. Crosby et al. indicated that for patients, the change in HRQoL may reflect the reduction in symptoms or improvement in function, whereas for doctors, the change may reveal the effectiveness of the treatment or in the prognosis of the illness [46]. The MID estimate could help us to understand what change in index score is clinically meaningful from a comprehensive perspective. Our study found the MID estimate for our sample of HK’s general population is higher than the MID of other jurisdictions [45], which may illustrate that a higher price our social care system has to pay for improving the local people’s health. The MID could be considered as a threshold effect providing information that a clinical treatment could not be indicated and offers the most direct considerations for both benefits and harms from the patient perspective [47]. Given these values have clinical and social implications in defining and comparing the people’ health states, MIDs for different conditions, especially chronic conditions, should be estimated. Thus, we will report our MID estimation using EQ-5D index values for different physical and mental conditions in the following studies.

One strength of using EQ-5D is that the specific HK EQ-5D-5L value set is well-established, thereby overcoming some cultural biases that might arise when using other jurisdiction’s values. Another strength is that our results could be used as a baseline to conduct the economic evaluation in HK and ensure consistency with evidence in the future studies. In addition to the strengths already mentioned above, there are some limitations worth noting. First, the ceiling effect was more than 45% in our study, which could influence the precision of calculating quality-adjusted life years in certain economic evaluations. Another one is based on cultural considerations, in that, we did not include the actual income as a variable into our survey, and instead used employment and living status as surrogate variables; actual income may be an important factor influencing QoL. Also, the sample size of patients in our study who reported having multimorbidity was not big, and thus a large population-based multimorbidity survey is needed in future studies. The last limitation is that chronic conditions in our study may not have had adequate precision, given that they were self-reported by individuals and possibly influenced by recall bias. In future studies, we suggest using the ICD-10 codes of the International Classification Diseases to define health conditions.

## Conclusion

This study explored the burden of chronic conditions and multimorbidity on HRQoL and primarily defined the MID estimate using EQ-5D-5L in HK’s general population. The findings suggest that HRQoL was negatively affected by the chronic conditions of physical disabilities, mental problems, and hypertension, as well as multimorbidity, all of which were strongly associated with low HRQoL. Reforming the healthcare system address foreseeable challenges arising as more patients live with chronic conditions and multimorbidity could improve service efficiency and the effectiveness of healthcare interventions and policies, and, ultimately, improve the HRQoL of HK citizens.
